# Reset-Voltage Controlled Resistance-State and Applications of Forming-Free Fe-Doped SrTiO_3_ Thin-Film Memristor

**DOI:** 10.3390/ma17205021

**Published:** 2024-10-14

**Authors:** Ke-Jing Lee, Cheng-Hua Wu, Cheng-Jung Lee, Dei-Wei Chou, Na-Fu Wang, Yeong-Her Wang

**Affiliations:** 1 Center for Environmental Toxin and Emerging-Contaminant Research & Technology Center, Department of Electronic Engineering, Cheng Shiu University, Kaohsiung City 83347, Taiwan; 2227@gcloud.csu.edu.tw (D.-W.C.); k0481@gcloud.csu.edu.tw (N.-F.W.); 2 Institute of Microelectronics, Department of Electrical Engineering, National Cheng-Kung University, Tainan 701401, Taiwan; q16091128@gs.ncku.edu.tw (C.-H.W.); 11201043@ncku.edu.tw (C.-J.L.)

**Keywords:** resistive random-access memory, sol–gel, strontium ferrite titanate

## Abstract

In this study, we prepared a strontium ferrite titanate (STF) thin film using a sol–gel process to insulate resistive random-access memory (RRAM) applications. Compared to the typical strontium titanate (STO) RRAM, the improvement in the resistive switching characteristics in STF RRAM is obvious. The Al/STO/ITO/Glass RRAM set/reset voltages of −1.4 V/+3.3 V and the Al/STF/ITO/Glass RRAM set/reset voltages of −0.45 V/+1.55 V presented a memory window larger than 10^3^, a low operating voltage and device stability of more than 104 s. In this study, the influence of Fe on the conducting paths and the bipolar resistive switching properties of Al/STF/ITO/Glass RRAM devices is investigated.

## 1. Introduction

In the electric power industry, the demand for intelligent electronic devices has quickly increased and highly promoted the development of the Internet of Things (IoT). As electronic devices become faster and faster, they must handle storing large amounts of data. Hence, memory with low power dissipation, high storage density, excellent scalability, and high reliability (retention and endurance) is essential. In recent years, researchers have spent a long time controlling the distribution and density of oxygen vacancies through various process steps and structures. In the case of oxygen ions within anodic crystalline HfO_2_, detected anodic photocurrent was attributed to optical transitions between allowed electron localized states in the oxide band gap and the conduction band, accompanied by photoelectrochemical and electrochemical redox reactions near the Pt/HfO_2_ interface, resulting in bipolar resistance switching [1,2,3,4,5,6,7,8].

SrTiO_3_ thin films deposited via a sol–gel method have been presented [9,10], and the sol–gel process perovskite device shows advantages such as low cost, excellent scalability, and fast operation speed. Among the main reasons for studying SrTiO_3_ are its chemical defects, including oxygen diffusion, tuned electron defects, and cationic diffusion, which have led it to become a model material for a wide range of applications in memory due to the systematic change in its physical properties with oxidation state and doping [11,12,13]. Its physical properties are susceptible to systematic changes with oxidation state and doping, and thus may exhibit many excellent electronic properties [14,15].

Based on the fact that the transmission in STO is typically and mainly related to extended defects, here we could assume that filament formation is influenced by Fe doping [16]. Previous research has presented intriguing characteristics such as oxygen-sensitive, photochromic, and ferroelectric properties. Additionally, defect state, dielectric properties, as well as conduction mechanism have also been investigated. Compared to the common SrTiO_3_, defect states such as oxygen vacancies of strontium ferrite titanate (STF) support charge balancing in view of the various valence states regarding Fe and Ti ions. Experimentally and theoretically [17], oxygen vacancies are the controlling defect for Fe-doped STO and clustering around Fe atoms in the first coordination shell [18]. In addition, Fe ion doping was reported to regulate the band gap of SrTiO_3_. In summary, Fe ion doping can enhance oxygen vacancy conductive filament origination in SrTiO_3_ films, thus remarkably reducing switching voltages [19]. Menesklou et al. found that, to maintain charge balance, doping Fe^3+^ to replace Ti^4+^ in the STO film creates oxygen vacancies, thus further increasing the switching ratio [20]. It indicates promising applications for low operating voltage memory devices.

In this study, STO/STF films were produced via the sol–gel approach to investigate their resistive switching properties. We discuss electrical properties such as I-V curve, uniformity, and reliability issues. Additionally, several physical analyses were also conducted, including crystal structure and composition of films, surface morphology, and thickness.

## 2. Results and Discussion

**Figure 1:**
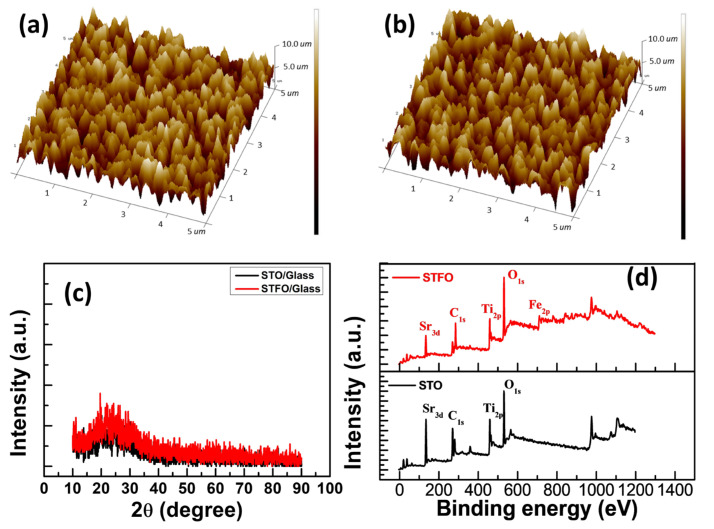
The surface morphologies of (**a**) STO, and (**b**) STF thin films inspected by AFM. (**c**) XRD pattern of STO and STF thin film. (**d**) XPS full spectra analysis of the STO, and STF thin films.

The atomic force microscopy (AFM) analyses performed on samples by STO and STF on surface morphology before and after Fe ion doping are presented in Figure 1a and Figure 1b, respectively. The values of the root mean square surface roughness measured for specific profiles of STO and STF samples are 1.74 and 1.16 nm, respectively. Roughness does not significantly change after Fe ion doping [21]. To prove the crystal structure of STO and STF thin films, an X-ray diffractometer (D8 DISCOVER with GADDS (Bruker AXS Gmbh, Karlsruhe, Germany)) pattern was employed to distinguish the structure of the films. The XRD results of STO and STF are depicted in Figure 1c. As shown in the analysis results, no obvious peaks were observed in the XRD spectrum. The results showed that the thin films prepared in this study were amorphous regardless of Fe ion doping. Additionally, the broad peak near 25° was ascribed to the glass substrate [22].

Figure 1d shows the X-ray photoelectron spectroscopy (XPS(ULVAC-PHI PHI VersaProbe 4)) patterns obtained after 1 min 30 s of Ar^+^ ion sputtering, thereby representing the bulk layer of the STO and STF thin films. In he STO thin film, the atomic percentages of Sr, Ti, and oxygen (O) were obtained at 19.1%, 18.5%, and 62.4%, respectively. The high-resolution Sr 3d spectrum showed that the peaks of Sr 3d_5/2_ and 3d_3/2_ correspond to two peaks at ~132 eV and ~134 eV, respectively. The binding energy of Ti is approximately 463.9 eV and 458.2 eV, corresponding to two peaks at Ti 2p_1/2_ and Ti 2p_3/2_, respectively. These peaks correspond to the Ti^4+^ oxidation state. The STF thin film and the atomic percentage of Sr, Ti, Fe, and O were obtained at 11.2%, 9.3%, 8.9%, and 70.6%, respectively. The Fe 2p core level has been fitted with two nearly Gaussian components. The peaks located at 708.9 and 710.4 eV can be assigned to Fe^2+^ and Fe^3+^, respectively.

**Figure 2:**
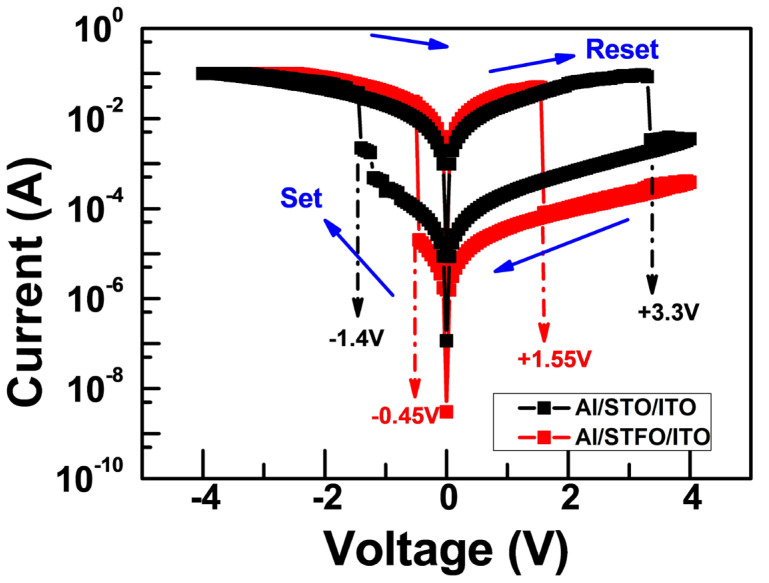
I-V characteristics of Al/STO/ITO and Al/STF/ITO RRAM device.

The I-V curves of STO and STF RRAM devices were measured by a Keysight B1500A parameter analyzer. A bias was applied on both an Al top electrode and an ITO bottom electrode grounded with a compliance current limiting device current below 100 mA. Figure 2 depicts the typical I–V curves of Al/STO/ITO and Al/STF/ITO RRAM devices. Furthermore, the bipolar and reproducible resistive switching behaviors of the devices were also observed. The current increased abruptly at −1.4 V and −0.45 V for the corresponding devices during the negative sweep. This process is regarded as “set”, which indicates the resistance state from HRS to LRS. Subsequently, during the positive sweep, a pronounced decrease in the current could be observed at +3.3 V and +1.55 V for the corresponding devices. This process is regarded as “reset”, which indicates the transition of the resistance state back to HRS. Compared with the STO device, the reset/set voltage of the STF device exhibits significant performance. Both reset/set voltage decreased significantly. A high switching ratio (R_off_/R_on_) of 10^3^ was demonstrated in the Al/STF/ITO RRAM device. The ON/OFF ratio of Al/STO/ITO RRAM device was maintained above 10^2^.

**Figure 3:**
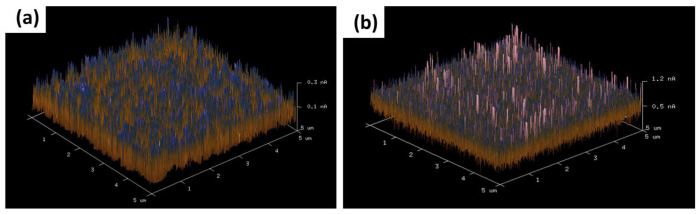
C-AFM (5 × 5 μm^2^ area) measurement of (**a**) STO and (**b**) STF structure.

To acquire further information on the conducting mechanism in both structures, atomic force microscopy (CAFM) measurements were performed directly to clarify the resistive switching behavior in STO and STF thin films. The formation of the conducting paths in STO and STF films were as large as 0.3 and 1.2 pA in Figure 3. In addition, the CAFM image of the STF film exhibits abundant current peaks with higher area density compared with the STO film. Because of these results, we propose that the added iron element can offer more opportunities to generate conducting paths in the switching process. Therefore, high device yield and reproducible switching behavior obtained from the Al/STF/ITO/Glass structure are expected.

**Figure 4:**
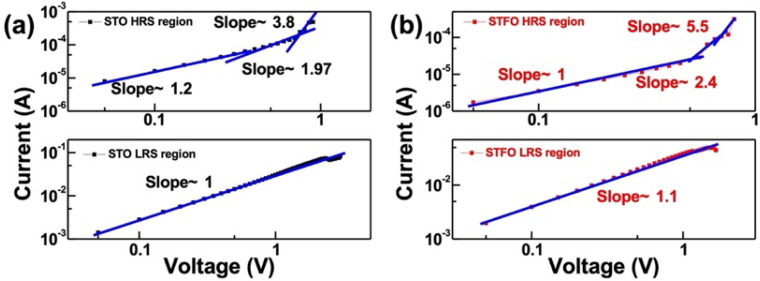
LogI–logV plots of (**a**) STO device and (**b**) STF device at LRS and HRS.

To determine the process of the resistive switching inside STO and STF RRAM, curve fittings on I-V were performed after the devices were fabricated. Figure 4 presents the fitting results of STO and STF devices at LRS in a log–log scale, respectively. The fitting results of the STO and STF devices at the LRS region exhibit ohmic conduction behavior (slope ~1), which confirms the formation of conductive filament in the device can be checked by the linear dependence of device current versus applied voltage. HRS with a slope of 1 at low voltage conditions indicates ohmic conduction (I∝V^1^). With increases in the applied voltage, more and more electrons are injected into the insulating layer, leading to a disproportion in the space charge. The slopes of the STF devices fitted in the Child’s law region (I∝V^2^) as 2.4, and increased to 5.5 at the tail, which are much higher values than those of the STO devices shown in the left. This result apparently shows that the current in HRS is dominated by the space charge limited current (SCLC) conduction inside [23,24,25]. These conduction behaviors collectively resulting from the resistive switching mechanism can be further factored as the formation and rupture of filaments near the interface. Additionally, in the STF, higher slope devices indicate better conductivity in the STF film.

**Figure 5:**
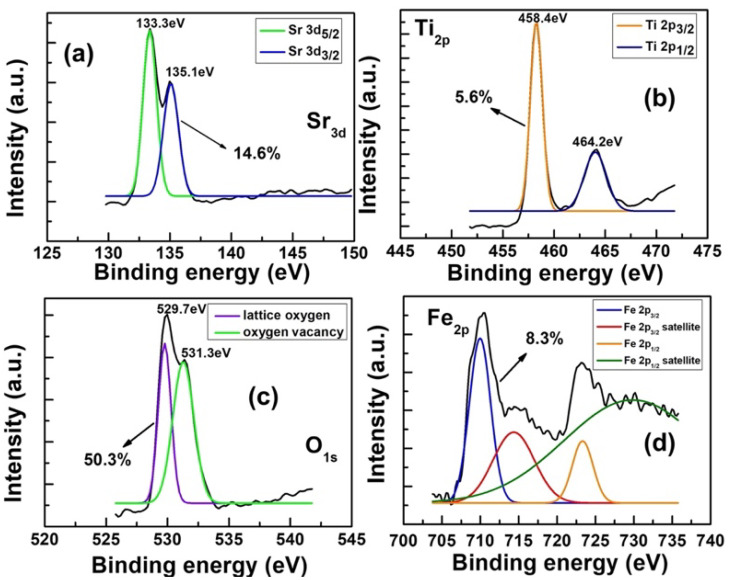
XPS narrow-scan spectra for all compositions in the surface of SFT thin film of (**a**) Sr 3d (**b**) Ti 2p (**c**) O 1s and (**d**) Fe 2p.

The spin energy separation is approximately 1.8 eV between two peaks at 133.30 eV for Sr 3d_5/2_ and 135.1 eV for Sr 3d_3/2_, as shown in Figure 5a. The high-resolution core level spectra of Sr 3d indicates that Sr ions are in the 2 + chemical state [26]. Figure 5b shows that the Ti 2p_3/2_ and 2p_1/2_ peaks are located at 458.4 eV and 463.80 eV, respectively. The spin energy separation is 5.4 eV, suggesting the presence of Ti with + 4 state. The suspicious peaks of Sr 3d, Fe 2p, and Ti 2p may come from the doublet form of the O 1s spectrum, matching the assumption that there are mixed oxides for some components in SFT thin film. Figure 5c shows the XPS results for the surfaces of the STO film and STF film. The O 1s peak transformed into two peaks at 529.7 ± 0.2 eV and 531.3 ± 0.3 eV by Gaussian fitting, which agrees with the results reported in amorphous SrTiO_3_ [9]. The peak at 529.7 ± 0.2 eV is assigned to the well-bonded oxygen lattice bond with the cation, while peaking at 531.3 ± 0.3 eV is assigned to the oxygen vacancy bond. According to XPS results, the O_vacancy_/O_lattice_ ratio, where O_lattice_ is lattice oxygen, can be used as a factor for calibration in the ratio with the oxygen-vacancy bond decreasing from 2.02% to 1.47% as a result. A study by Huang et al. has shown that the amount of filament paths is related to the density of oxygen vacancy, thus the high amount of oxygen vacancies will lead to the sudden formation of randomly numerous filaments and further affect the performance and stability of the memory devices in amorphous thin film [27,28,29]. The HRS currents and reset voltages of the STO memory devices are relatively higher than those of the STF memory device. The XPS results show the trend of oxygen vacancy and further explain the lower HRS current or higher on/off ratio of the STF RRAM.

Furthermore, the XPS narrow scan for Fe 2p in Figure 5d involves the prominent peaks of Fe 2p_3/2_, Fe 2p_1/2_, and satellites. The Fe 2p_3/2_ peak is located at 709.80 eV, and the sensitive satellite of the Fe 2p_3/2_ peak is located at 714.00 eV. The Fe 2p_1/2_ and its satellite peak at 722.7 and 729.3 eV, respectively. The intensity of the Fe 2p_3/2_ peak is stronger than the Fe 2p_1/2_ due to the spin-orbit coupling [30]. Corresponding to the doublet of Fe 2p on the spectrum, two valence states of Fe in SFT thin film are presented as Fe^2+^ and Fe^3+^. The dominated Fe^3+^ is one of the significant contributions to oxygen vacancies generation.

**Figure 6:**
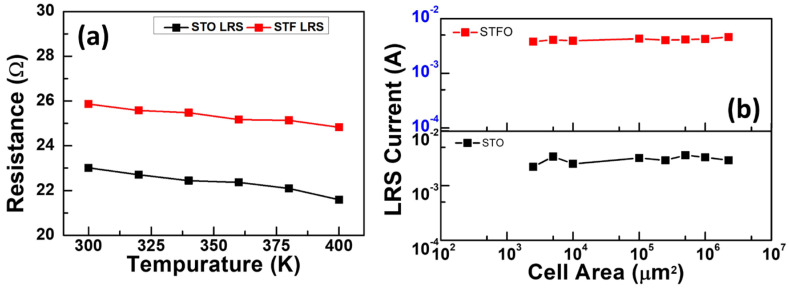
(**a**) Temperature-dependent resistance of STO and STF RRAM device measured between 300 K and 400 K. (**b**) LRS current versus cell area of STO and STF memory devices.

Figure 6 depicts the resistance at the LRS and HRS, both as a function of temperature in the range from 300 K to 400 K. The I-V curves were measured via the thermal rising system, and the samples were heated from 300 K (room temperature). After that, they were measured for every 20 K rise in temperature until 400 K. As shown in Figure 6a, we could detect that the resistance of LRS is inversely proportional to the temperature, showing semiconductor behavior. Modulable conductivity of the oxide by accumulated number of oxygen vacancies in the oxide/top electrode interface, can be viewed as semiconductor behavior in LRS. As a result, the switching mechanisms of the STO and STF both count on the oxygen vacancy migration. The dependence of LRS resistance on the device area of the STO and STF memory devices is presented in µ6b. Consequently, it has no positive relativity to the cell area, which shows that, in the device, LRS current is mainly filamentary [31].

**Figure 7:**
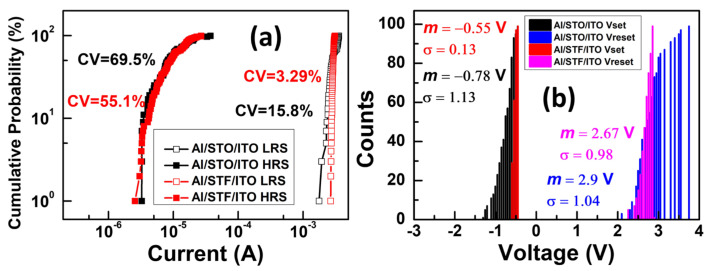
Statistical distributions of (**a**) current values and (**b**) voltage values of Al/STO/ITO and Al/STF/ITO structure.

To confirm the uniformity issues regarding the comparison of STO and STF RRAM devices, the statistical distribution of the operation voltage and current was investigated. Figure 7a. shows the LRS/HRS current distribution of STO and STF RRAM devices. The average (AVG) and standard deviation (STD) of the LRS/HRS current of STO RRAM device were AVG of 2.9 × 10^−3^ /1.07 × 10^−5^ A and STD of 4.58 × 10^−4^/7.44 × 10^−6^ A. The coefficient of variation (CV) is defined as the ratio of STD to AVG. On the other hand, the AVG and STD of the LRS/HRS current of the STF RRAM device were AVG of 2.9 × 10^−3^ /1.04 × 10^−5^ A and STD of 9.61 × 10^−5^/5.76 × 10^−6^ A. Hence, the CV of the LRS/HRS current of STO and STF RRAM devices are 15.8%/69.5% and 3.29%/55.1%, respectively. According to the results, the distribution of LRS current is more concentrated compared to the HRS current, and it indicates that reducing the HRS current is a significant issue for RRAM operation. In addition, compared with the STO RRAM device, the STF RRAM device shows slightly better uniformity of both LRS and HRS. Figure 7b depicts the operation voltage distribution of STO-based and STF-based RRAM devices. The AVG and STD of the set/reset voltage of STO-based RRAM device were AVG of −0.78/2.78 V and STD of 0.22/0.35 V. Then, the AVG and STD of the set/reset voltage of STF RRAM device were AVG of −0.58/2.95 V and STD of 0.11/0.37 V. Therefore, the CV of the set/reset voltage of STO and STF RRAM devices are 28.06%/12.74% and 19.17%/12.45%, respectively. We could observe that the set voltages demonstrate tighter voltage distribution compared to reset voltages. Furthermore, the set voltages of the STF RRAM device reduce remarkably compared to the STO RRAM device. Both of devices exhibit a similar performance of reset voltage distribution. In summary, the STF RRAM device has excellent current and voltage distribution, indicating high uniformity.

**Figure 8:**
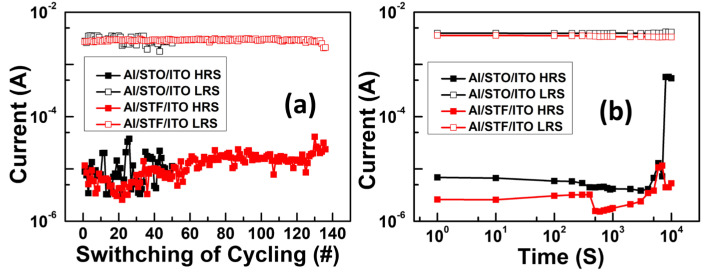
(**a**) DC cycle endurance and (**b**) retention characteristics of STO and STF RRAM device at 0.1 V under room temperature.

The DC cycle endurance test of the STO and STF RRAM device is presented in Figure 8a, in which the current was measured at 0.1 V. According to the results, we can observe that the highest DC cycle endurance of the STO RRAM is about 50 cycles. However, the highest DC cycle endurance of the STF RRAM is over 135 cycles with no significant degradation. On the other hand, the STO RRAM device suffers from severe HRS current fluctuation, while the current of the STF RRAM device is more stable. The statistical data of the endurance test exhibited the steady resistive switching operation and ensured that the switching between OFF and ON states could be reproducible and reversible. In Summary, the STF RRAM device shows high stability in terms of its resistive switching characteristic. To assess the capability of data retention of the STO and STF RRAM devices, retention characteristics measured at room temperature were executed in Figure 8b. First, a negative sweep was applied on the devices to activate the set process and switch the resistance state from HRS to LRS. Subsequently, the read bias at 0.1 V was used to record the current in each second until failure occurred. Similarly, when the reset process was triggered, identical read bias was used to record the HRS current. The retention time of the STO RRAM device was longer than 10^3^ s, whereas the HRS degraded abruptly after 7000 s. In contrast, The STF RRAM device was longer than 10^4^ s. In addition, the retention memory window of 10^3^ was attained without noticeable degradation at room temperature. According to the results, the STF RRAM device exhibits remarkable environmental stability.

**Figure 9:**
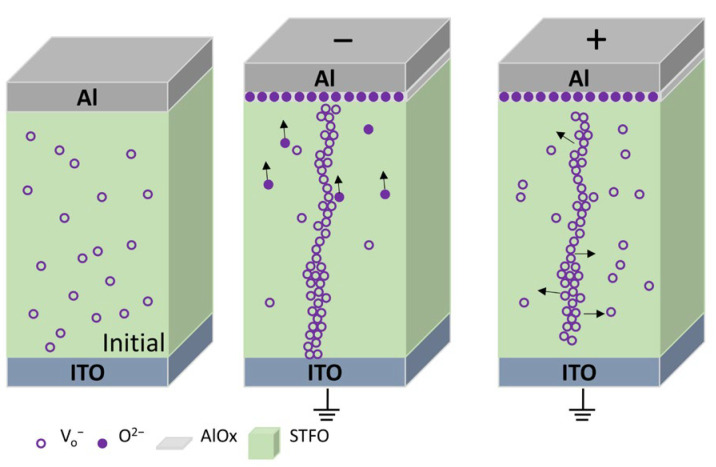
Resistive switching mechanism model of the STF-based RRAM.

As Figure 9 shows, in the initial state of Al/STF/ITO device, when a negative bias is applied on the top electrode, the oxygen ion in the oxide moves toward the TE, leaving an oxygen vacancy, and the positively charged oxygen vacancy is released to form the conductive filament (this is the SET process). Contrarily, whenever the top Al electrode is positively biased, O^2−^ moves in the opposite direction, while the filament dissolves due to the redox reaction and sets the device from HRS to LRS. For the Al/STO/ITO conduction mechanism [9], which occurred in STO thin film, the formation and rupture of the conduction filament had a certain randomness, which led to a certain fluctuation during operation (Figure 2). Fe-doped STO thin film effectively reduces such randomness, whereas STF film can act as an oxygen ion reservoir.

## 3. Conclusions

In conclusion, resistive switching behaviors in sol–gel strontium ferrite titanate thin films have been probed under low processing temperature. By doping Fe ion into the STO to generate the STF quaternary metal compounds prepared via the sol–gel method, excellent resistive switching characteristics of Al/STFO/ITO/Glass RRAM have been demonstrated. Without any surface treatment or high temperature annealing, the STF-based RRAM shows good uniformity, including a stable retention time of more than 10^4^ s, a resistance ratio larger than 10^3^ and passable endurance cycles. The conduction mechanisms of LRS and HRS are dominated by Ohmic conduction and Space-charge-limited conduction, respectively. The results revealed that the set/reset voltage could be reduced effectively by regulating the bandgap of insulators. Hence, using composite material is said to be a feasible method to modify the performances of RRAM devices.

## 4. Materials and Methods

**Figure 10:**
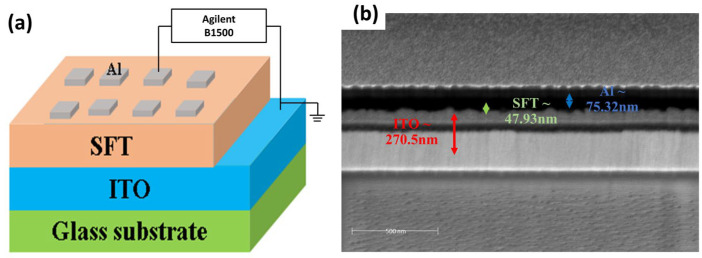
(**a**) Schematic of the Al/STF/ITO device, (**b**) FIB cross-section image of the fabricated device.

Experimentally, the strontium ferrite titanate (STF) solution was prepared in three steps. First, Strontium acetate (268.4 mg) was dissolved in glacial acetic acid (3.2 mL) by stirring, then heated on a 100 °C hot plate until fully dissolution (Z_1_ solution). Next, Iron acetate (217.4 mg) and Titanium isopropoxide (380 μL) were mixed with acetylacetone (470 μL) and dissolved in 2-methoxyethanol (3.2 mL) by stirring on a 100 °C hot plate (Z_2_ solution). Finally, Z_1_ was slowly dropped into Z_2_ until a completely transparent solution was obtained. Then, The STF solution was spin-coated under 500/5000 rpm for 5/15 s onto the ITO/glass substrate. After that, the sample was baked in a vacuum oven at 100 °C for 10 min under ambient air conditions. Lastly, the Al electrode was deposited by the DC sputtering system at 0.25 mm^2^ using a shadow mask for electrical measurements. Figure 10a shows the schematic of the fabricated device. Figure 10b shows the FIB cross-sectional image of the fabricated device.

## Data Availability

All data generated or analyzed during this study are included in the present article.
